# Open triple-branched stent graft placement for the surgical treatment of acute aortic arch dissection

**DOI:** 10.1186/1749-8090-7-130

**Published:** 2012-12-15

**Authors:** Xiaoning Sun, Shuyang Lu, Shouguo Yang, Hao Lai, Hao Chen, Tao Hong, Chunsheng Wang

**Affiliations:** 1 Department of Cardiac Surgery, Zhongshan Hospital, Fudan University, 180 Fenglin Road, Shanghai, 200032, China

**Keywords:** Acute Aortic dissection, Aortic arch, Triple-branched stent graft

## Abstract

**Background:**

The primary experience of open triple-branched stent graft placement for acute aortic arch dissection was reported.

**Methods:**

Between January 2011 and October 2011, 13 well-selected patients (mea age, 46±8.2 years; approximate range, 30~58 years) with acute aortic arch dissection underwent open triple-branched stent graft placement for total arch reconstruction. The triple-branched stent graft was a branched 1-piece graft consisting of a self-expandable nitinol stent and polyester vascular graft fabric (Yuhengjia Sci Tech Corp Ltd, Beijing, China).During hypothermic circulatory arrest, through the transverse incision of the ascending aorta, the main graft of the triple-branched stent graft was inserted into the true lumen of the arch and proximal descending aorta, and then each sidearm graft was positioned one by one into the aortic branch. Once the main graft and sidearm grafts were properly positioned, the restraining strings were withdrawn and then the main graft and sidearm grafts were deployed. Enhanced electric beam computed tomography was performed in each patient before discharge to evaluate the postoperative time course of the residual false lumen.

**Results:**

Open triple-branched stent graft placement was technically successful in all patients. The mean cardiopulmonary bypass time, aortic cross-clamp time and arrest time were 138.40±47.75 min, 70.60±28.94 min and 28.60±12.48 min, respectively. All patients were discharged from hospital. Their computed tomographic scans postoperatively showed that all stent grafts were fully opened and not kinked, there was no blood flow surrounding the triple-branched stent graft.

**Conclusion:**

Open triple-branched stent graft placement is a new effective technique for total arch reconstruction in acute arch dissection.

## Background

The conventional elephant trunk technique has been widely used for staged aortic replacement [1-3]. Crawford and associates [4] reported the complications of this method: kinking and occlusion of the graft, paraplegia as a result of clotting around the graft, and peripheral thromboembolism caused by flapping action. To avoid these potential complications of a long elephant trunk graft, Kato et al. [5] inserted a graft, the distal part of which was sustained by an intraluminal stent into the descending aorta. Thus any kinking and flapping action of the graft could be prevented. In addition, the dead space around the graft could be completely obliterated. Sun et al. [6] used this method and made some modifications: the whole length of the elephant trunk is sustained by a self-expandable stent, and this is the so-called “skeletonized elephant trunk technique”. Ascending aorta and total arch replacement combined with transaortic stent graft implantation has been successfully used in the treatment of acute arch dissection, Therefore, this hybrid technique has been accepted as the preferred surgical treatment for acute arch dissection when extensive thoracic aorta repair is needed [7-9]. However, the hybrid technique [5,6] of Kato or Sun’s is still a complex procedure, mainly because it still requires careful manipulation of the arch and elaborate anastomosis to the distal arch and three arch vessels.

In an effort to simplify extensive primary repair of the thoracic aorta, Shimamura [10] developed a two branched endoprosthesis to reconstruct the descending aorta and cervical branches simultaneously in a single circulatory arrest period through the opened proximal aortic arch. Chen et al. [11] developed a triple-branched stent graft (branched 1-piece stent graft) placement technique in which extensive primary repair of the thoracic aorta could be performed simply by both open placement of the triple-branched stent graft into the proximal descending aorta, arch, and three arch vessels and graft replacement of the ascending aorta. We describe the initial clinical results of our application of this new technique in 13 patients with acute arch dissection.

## Methods

### Patients

The subjects were 13 patients (12 men and 1 women) with acute Stanford type A aortic dissection involving the aortic arch, they underwent extensive primary repair of the thoracic aorta by means of ascending aorta replacement combined with open placement of triple-branched stent graft from January 2011 and October 2011.The ethics committee of our Hospital approved the procedure. We obtained written informed consent from each patient. The average age was 46±8.2 years; approximate range, 30~58years. The diagnosis was based on electricbeam computed tomography and echocardiography. The 13 patients were selected for extensive primary repair of the thoracic aorta by means of ascending aorta replacement combined with open placement of triple-branched stent graft because they satisfied following inclusion and exclusion criteria. The indications were acute Stanford type A aortic dissection with (1) the intimal tear located in transverse arch or proximal descending aorta that could not be resected by hemiarch replacement, (2) serious involvement of the arch vessels.

The 13 patients selected for triple-branched stent graft placement satisfied the additional criteria [11]: (1) the diameters of the native aortic arch and arch branches were 10-20% smaller than those of the corresponding stent grafts, (2) the distances between two neighbouring arch branched were equal to the distances between two corresponding sidearm stent grafts, (3) no history of carotid artery disease, (4) no aortic aneurysm in arch or proximal descending aorta and (5) all three arch branch ostia could be seen clearly from the arch true lumen through the transverse incision of the distal ascending aorta. The positions and the diameters of the native aortic arch and arch branches are different in different cases; we measured these data by preoperative three-dimensional computed tomography and then chose the optimal size for individuals.

### Description of the device

**Figure 1:**
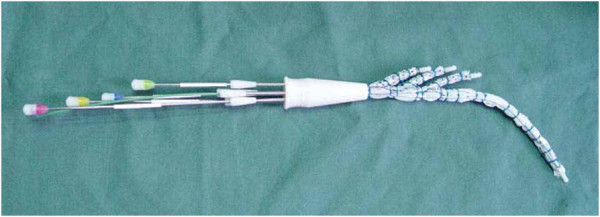
The main graft and 3 sidearm grafts were individually mounted on 4 catheters and restrained by 4 silk strings.

The triple-branched stent graft is a branched 1-piece graft consisting of a self-expandable nitinol stent and polyester vascular graft fabric (Yuhengjia Sci Tech Corp Ltd, Beijing, China). It comprised a main graft and 3 sidearm grafts. The main graft was tapered and flexible enough to conform to the curved aortic arch. The tapered main graft was 145 mm in length, 30 mm in proximal diameter, and 26 mm in distal diameter. At its proximal end, there was a 10-mm-long stent-free sewing Dacron tube. The first sidearm graft was 35 mm long and 14 or 16 mm in diameter. Both the second and third sidearm grafts were 25 mm long and 12 or 14 mm in diameter. The distance between 2 neighboring sidearm grafts was 3 mm. The main graft and 3 sidearm grafts were individually mounted on 4 catheters and restrained by 4 silk strings (Figure 1).

### Surgical technique

**Figure 2:**
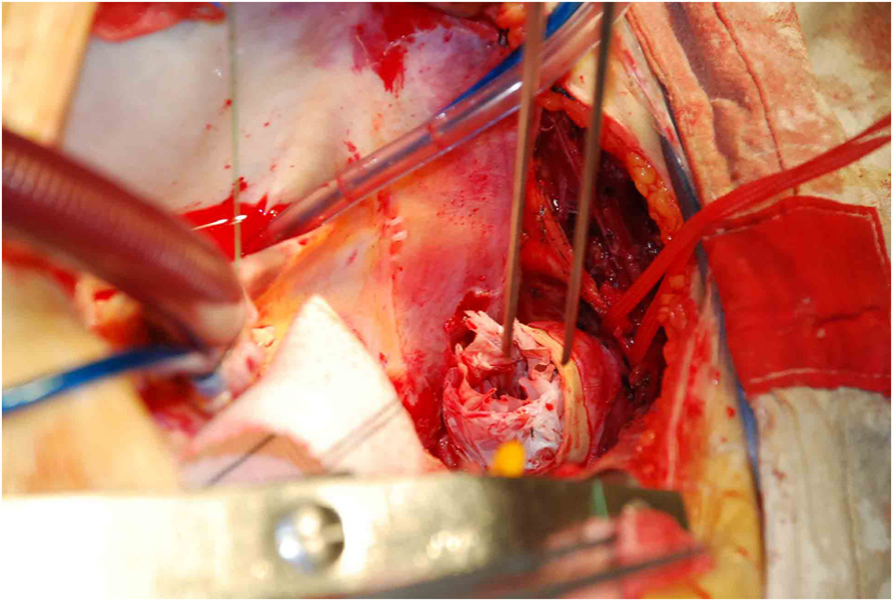
Introperative view after the grafts were deployed.

All procedures were performed with patients under general anesthesia. The arterial blood pressure of both the upper and lower limbs was monitored, and a probe for transesophageal echocardiographic monitoring was inserted. A median sternotomy was performed in all patients. cardiopulmonary bypass was established by 2 venous cannulas through the right atrium and 2 arterial return cannulas placed in the femoral and right axillary arteries. A left ventricular vent was inserted through the right superior pulmonary vein. Systemic cooling was initiated while the ascending aorta, aortic arch vessels, and descending thoracic aorta were exposed. The ascending aorta was clamped at the base of the innominate artery and transected just above the sinotubular junction. Cardioplegia was infused directly into the both coronary arteries. After the internal surface of the ascending aorta and the aortic valve were examined, proximal manipulations were performed. Then, the ascending aorta was anastomosed to a short segment of a straight graft (Hemashield Gold; Meadox Medicals, Oakland, New Jersey) Systemic cooling progressed until the patient’s nasopharyngeal temperature reached 20°C, and the pharmacologic agents (thiamylal sodium, phenytoin, and mannitol) were administered for brain protection [12] in addition to ice packs placed around the head, before circulatory arrest. After circulatory arrest had been instituted, the ascending aorta was unclamped and the aortic arch was opened. The innominate artery, left common carotid artery, and left subclavian artery were identified. Through the transverse incision of the ascending aorta, the main graft of the triple-branched stent graft was inserted into the true lumen of the arch and proximal descending aorta, and then each sidearm graft was positioned one by one into the aortic branch. Once the main graft and sidearm grafts were properly positioned, the restraining strings were withdrawn and then the main graft and sidearm grafts were deployed (Figure 2). Finally, after the release of the aortic arch stent, inserted into 40-degree saline gauze in the lumen, the shape memory alloy stent fully opened adherent, and 3–0 Prolene suture reinforced with Teflon band near the innominate artery, left subclavian artery and the main vessel stent through the suture arterial wall. The transected distal stump of the ascending aorta was reconstructed by the inner proximal stent-free Dacron tube of the main graft, and subsequently continuous anastomosis to the Dacron tube graft was made in an end-to-end fashion. The air was carefully flushed out from the triple-branched stent graft with femoral and right axillary blood return. Then, antegrade systemic perfusion from the branch of the Dacron tube graft was started, and the patient was rewarmed.

### Follow-up

All patients were contacted by telephone or direct interviews in our department after discharge. All patients followed up prospectively by means of contrast-enhanced computed tomography scan and general examination on the following schedule: before discharge, 3 months after the operation.

## Results

### Operative data

13 patients had hypertension without effective control. The primary intimal tears were located in the ascending aorta in 9 patients, in the arch in 3 patients, and in the proximal descending aorta with retrograde extension of the dissection into the arch and the ascending aorta in 1 patient. Placement of the triple-branched stent graft into the true lumen of the proximal descending aorta, arch, and 3 arch vessels was successful in all 13 patients and could be finished within 5 to 20 minutes. Concomitant procedures were Bentall operation in 2 patients, and coronary artery bypass in 1 patients. The mean cardiopulmonary bypass time was 138.40±47.75 minutes, aortic cross-clamp time was 70.60±28.94 minutes, and arrest time was 28.60±12.48 minutes.

We did not encounter any difficult bleeding from aortic anastomoses. No patient required additional surgery to correct excessive postprocedural bleeding. First 24h Drainage was 298±111 ml, There were no new cerebral deficits and no spinal cord injury postoperatively. Visceral malperfusion was not recognized. No acute renal failure required hemodialysis. No pulmonary complication resulted, and postoperative hoarseness did not occur in those patients. The postoperative mechanical ventilation support period was 22.0±13.67 hours. The duration of intensive care unit stay was 2.20±1.10 days. The operative variables are summarized in Table 1.

**Table 1:** Operative variables

**Variables**	
Operative data	
Cardiopulmonarybypass time (min)	138.40±47.75
aortic cross-clamp time (min)	70.60±28.94
deep hypothermia (min)	28.60±12.48
Early results	
First 24h Drainage (ml)	298±111
Ventalation Time (h)	22.0±13.67
ICU Time (d)	2.20±1.10
Hospital Time (d)	6.40±1.52
New cerebral complication	0
Renal failure requiring dialysis	0
Pneumonia	0

### Computed tomographic scans

**Figure 3:**
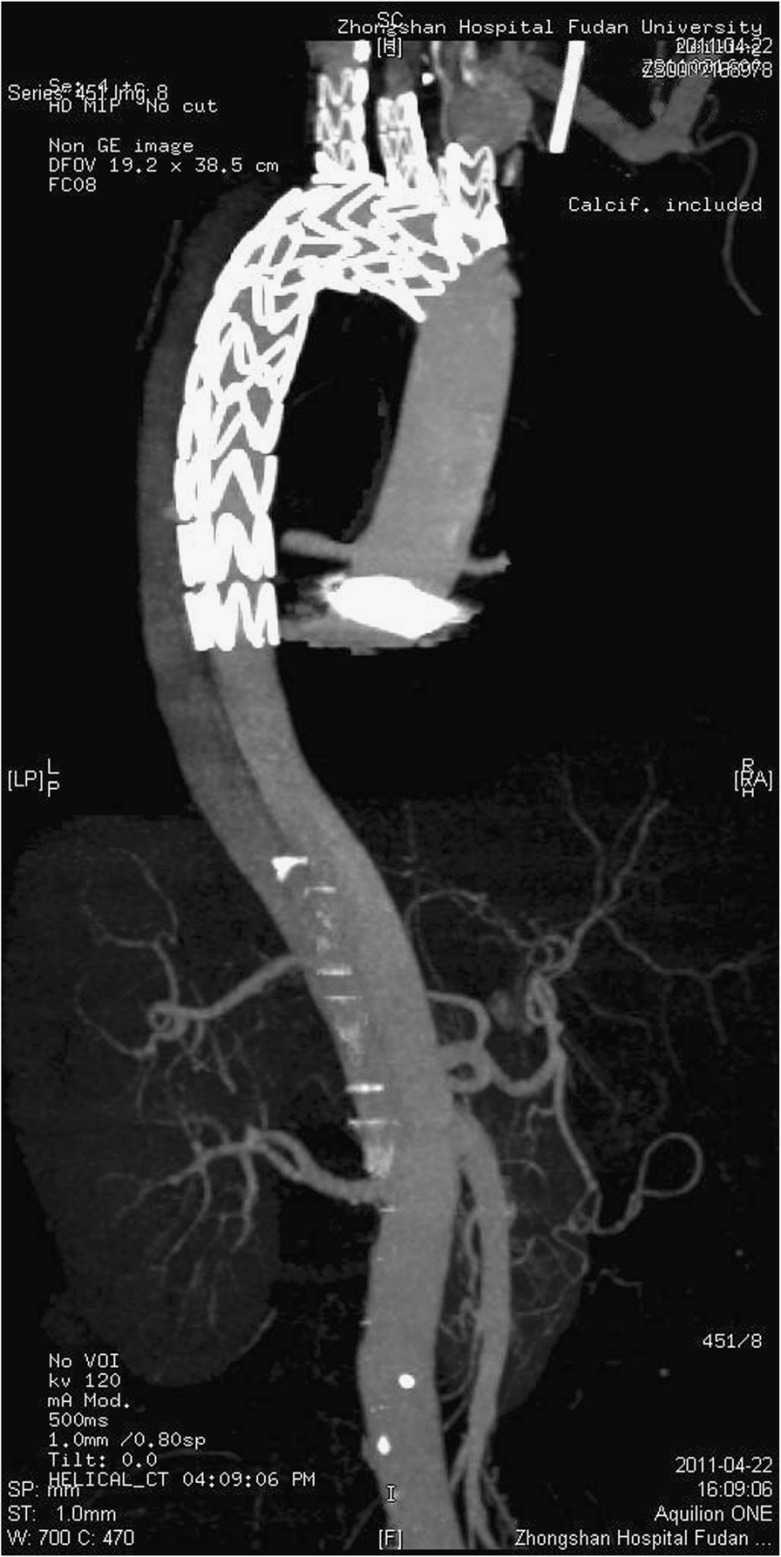
Angio CT Scan of the patient after surgical intervention shows that the true channel in the descending aorta was resumed, and the false channel was closed with thrombus.

**Figure 4:**
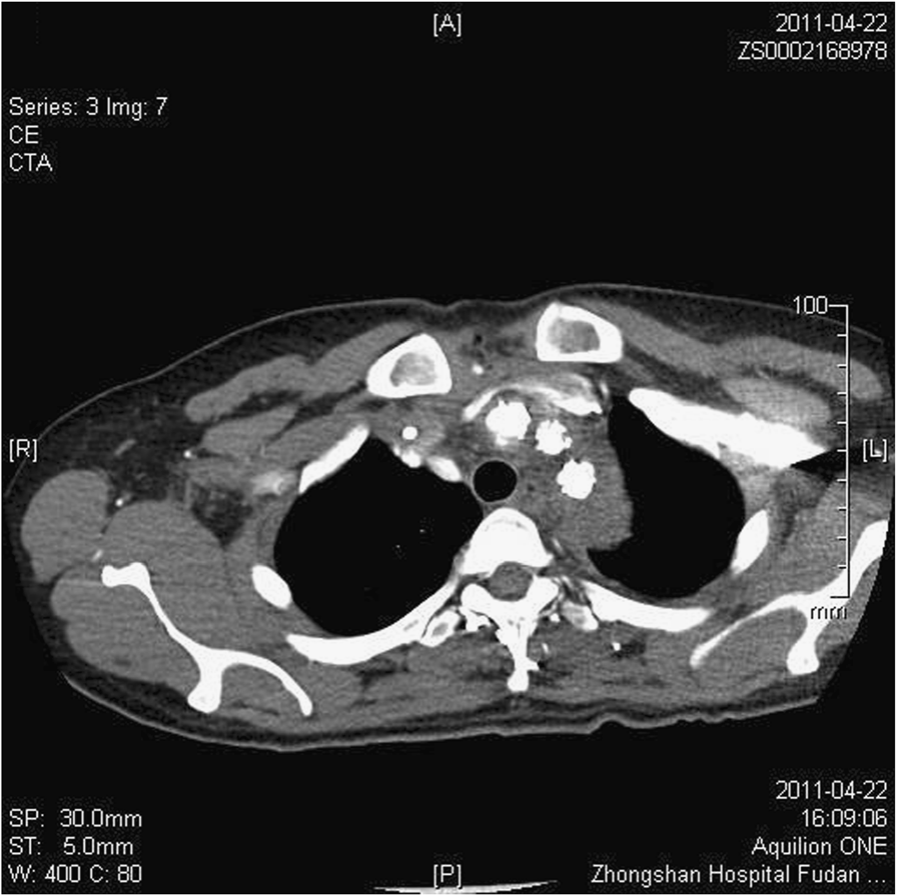
**Angio CT Scan shows that all stent grafts were opened and not kinked.** No sidearm graft occlusion was found.

The postoperative angio CT scans and echocardiographic examinations showed that all stent grafts were opened and not kinked, there was no space or blood flow surrounding the triple-branched stent graft. No sidearm graft occlusion was found (Figures 3,4).

### Follow-up

The follow-up was 100% complete. There were no late deaths and no need for reoperation during the mean follow-up of 9 ± 2 months (range, 3–12 months). No neurological complications were observed.

## Discussion

Endovascular stent graft placement has widely been confirmed as an effective aortic repair technique for acute aortic dissection, which can seal off the intimal tear and lead to quick clot formation and shrinkage of the false lumen [6,9].

The Shimamura’s [10] branched open stent-grafting technique is an evolutionary hybrid aortic arch repair procedure that combines conventional aortic surgery and endovascular repair with branched endoprosthesis. The outstanding point is that the branched open stentgrafting technique provides total arch repair without performing direct surgical reconstruction of the descending thoracic aorta and cervical branches. The distal aortic incision line is almost the same as in the hemiarch repair, and the branched endoprosthesis completes arch repair within an acceptably short interval of DHCA. But Shimamura’s endoprosthesis is homemade, it is not convenient to use. Our technique was designed to repair the proximal descending aorta, arch, and 3 arch vessels simultaneously by the simple open placement of triple-branched stent graft into the descending aorta, arch, and 3 arch vessels instead of direct surgical repair. In this study, we successfully applied our open triple-branched stent graft placement technique combined with graft replacement of the ascending aorta in extensive primary repair of the thoracic aorta in 13 patients with acute arch dissection. Placement of these triple-branched stent grafts into the descending aorta, arch, and 3 arch vessels was finished in 5 to 20 minutes during the procedure. All patients had recovered uneventfully and were discharged from hospital without complications. Their postoperative computed tomographic scans showed that all stent grafts were opened and not kinked, there was no endoleak and no sidearm graft occlusion.

Open placement of the conventional straight stent graft into descending aorta in the hybrid technique of Kato [7-9] has been proven to be an effective way of closing the residual false lumen of the descending aorta. Our preliminary results and others [11] demonstrated that the open triple-branched stent graft placement is a feasible and effective technique for extensive primary repair of the thoracic aorta in acute arch dissection.

The purpose of our technique is to obtain extensive primary repair of the thoracic aorta for acute arch dissection with less invasiveness. Our technique offers a number of advantages over the four-branched arch graft technique:

### First: less anastomosis, simplified the operation

In our technique, extensive primary repair of the thoracic aorta could be performed simply by both open placement of the triple branched stent graft into the proximal descending aorta, arch, and 3 arch vessels and graft replacement of the ascending aorta, which could reduce the risk and technical difficulties of extensive thoracic aorta repair to close to those of the conventional ascending graft replacement with open distal anastomosis. In the hybrid procedure of Kato [5], careful manipulation of the arch and elaborate anastomoses to the distal aortic arch and 3 arch vessels are time-consuming. Moreover performing anastomosis and hemostasis at the descending aorta is usually very difficult. Our open triple-branched stent graft placement technique can reduce such problems. Therefore, cardiopulmonary bypass time, aortic cross-clamp time, and selective cerebral perfusion and lower body arrest time were shorter than in the Kato and Sun’s hybrid technique seen in previous reports [5,6] and were comparable to those of conventional ascending replacement with open distal aortic anastomosis.

### Second: less bleeding

Open triple-branched stent graft placement technique makes it easy to control bleeding, we have observed less bleeding and did not encounter any difficult bleeding. No patient required additional surgery to correct excessive postprocedural bleeding. Three reasons explain this: first, anastomoses to 3 arch vessels were totally avoided; second, the distal aortic anastomosis at the distal ascending aorta, which provided a better surgical view for performing anastomosis and hemostasis; Finally, shorter time on cardiopulmonary bypass and deep hypothermic circulatory arrest contributed to the quick recovery of postoperative blood coagulability.

### Finally: less damage to vital organs and quick recovery

With our open triple-branched stent graft placement, as the surgical procedure simplified, the three branches of the aortic arch and descending aorta anastomosis reduced, deep hypothermic circulatory arrest time of significantly shortened, reducing the deep hypothermic circulatory arrest for vital organs such as brain, lung and kidney to avoid postoperative stroke, acute renal failure and dialysis. Reduce the adverse impact on lung function, avoiding the occurrence of acute respiratory insufficiency, reducing respiratory infection. Therefore, all patients had a short time on postoperative mechanical ventilation and a short stay in the intensive care unit and shortened hospital stay in this group. Improvement of prognosis after surgical repair of acute type A dissection is largely dependent on the reduction of complications.

Postoperative CT showed that all sidearm stent grafts were fully opened and no sidearm stent graft endoleak. For these good results, diameters of the 3 sidearm grafts, distances between 2 neighboring sidearm grafts, and proper placement of the triple-branched stent grafts were crucial. For each patient, the diameters of the selected grafts should be 10% to 20% bigger than the diameters of the native aortic arch and arch vessels, respectively [5,13]. The proper size of each graft was key for quick clot formation and shrinkage of the false lumen and for preventing new intimal trauma resulting from the continuous compression of the oversized stent graft on the dissected and frangible intimal wall [5,7]. The distances between 2 neighboring sidearm grafts should be approximately equal to the distances between 2 corresponding arch vessels, which would keep sidearm stent grafts from being twisted or kinked after deployment. It is reported that some risks such as postoperative stent graft shifting or kinking may be occur [14]. But in this study, no sidearm graft occlusion was observed because we use 3-0 Prolene suture reinforced with Teflon band near the innominate artery, left subclavian artery and the main vessel stent through the suture arterial wall to prevent the late shift and poor adhesion. The long-term patency of those sidearm grafts should be carefully evaluated.

## Conclusions

Open triple-branched stent graft placement is a simple and effective technique with satisfactory early results. With this technique, extensive primary repair of the thoracic aorta may become easier and safer for acute arch dissection. Careful long-term follow-up and further extensive clinical trials are necessary before this technique can become a recommended alternative to surgical extensive primary repair of the thoracic aorta for acute arch dissection.

## Abbreviations

CT: Computed tomography.

## Competing interests

The authors declare that they have no competing interests.

## Authors’ contributions

XS, CW participated in research design; XS, CW, SY, HL, HC, TH performed the experiments; XS, SL participated in the writing of the manuscript and indata analysis. All authors read and approved the final manuscript.
